# Genetic Variation of ITGB3 Is Associated with Asthma in Chinese Han Children

**DOI:** 10.1371/journal.pone.0056914

**Published:** 2013-02-22

**Authors:** Yan Zhang, Yuling Han, Liang Dong, Huafeng Yu, Lu Cheng, Xiuxia Zhao, Mingjie Ding

**Affiliations:** 1 Department of Respiratory Medicine, QiLu Children’s hospital of Shandong university, Jinan, Shandong, China; 2 Department of Respiratory Medicine, QiLu hospital of Shandong university, Jinan, Shandong, China; Democritus University of Thrace, Greece

## Abstract

Previous studies have demonstrated that integrins are involved in the aetiology of asthma. Several single-nucleotide polymorphisms (SNPs) in the integrin β3 (ITGB3) gene are significantly associated with asthma in Western populations. Given the important roles of environmental exposures in the development of asthma, we evaluated the associations between six SNPs in ITGB3 and asthma in Chinese Han children. A total of 321 unrelated Chinese children with asthma and 315 healthy children were recruited for the study. SNP genotyping was performed by high-resolution melting analysis (HRM). The selected SNPs were well genotyped by HRM, and SNP rs3809865 in the 3′ untranslated region (3′UTR) of ITGB3 was found to be strongly associated with asthma (adjusted *p* = 0.004). The minor allele of rs3809865 showed a protective effect against asthma (OR: 0.59; 95% CI: 0.43–0.8). The seed regions of two miRNAs (hsa-mir-124 and hsa-mir-506) were predicted to bind to the sequence containing rs3809865 by TargetScan and PITA. Luciferase reporter assays demonstrated that the T allele of rs3809865 was more efficiently targeted by hsa-mir-124 than was the A allele, which suggested that rs3809865 could affect the binding of hsa-mir-124 to ITGB3. Furthermore, the transfection of A549 cells with hsa-mir-124 resulted in the downregulation of ITGB3 expression. Our results revealed that rs3809865 was significantly associated with asthma due to its effect on the binding of hsa-mir-124 to ITGB3.

## Introduction

Asthma is one of the most common chronic diseases in children, and it is characterised by bronchial hyperresponsiveness (BHR) and reversible airway obstruction. Both genetic and environmental factors play important roles in the development of asthma. Although more than 100 genes have been associated with asthma, most of these associations have proven to be non-replicable in multiple populations, which indicates a complex genetic susceptibility pattern. Integrin β3 (ITGB3) is a serotonin-related gene on chromosome 17 that encodes a beta chain integrin subunit. Integrins are known to participate in cell adhesion and cell surface-mediated signalling. Recent investigations have suggested that ITGB3 is involved in the pathogenesis of asthma, particularly in early childhood [1], [2]. Five SNPs in the ITGB3 gene have been linked with asthma in a Hutterite population, but these results were not observed in three other unrelated populations, most likely due to differences in environmental exposures in childhood [2]. Rogers *et al*. demonstrated that few identified SNPs could be replicated in different populations [3]. Factors such as the criteria used to diagnose asthma, environmental exposures, numbers of subjects, different patterns of linkage disequilibrium, and population stratification could be potential causes of this non-repeatability [3], [4]. Multiple studies in diverse populations would be helpful to identify true candidate genes [3]. To date, few studies have been performed to investigate associations between the ITGB3 gene and asthma in Chinese Han children. Thus, it is necessary to identify single nucleotide polymorphisms (SNPs) in the ITGB3 gene associated with asthma in a Chinese population.

In the present study, we investigated the association of SNPs in ITGB3 with asthma in Chinese Han children using HRM analysis for SNP genotyping. Our study revealed significant associations between polymorphisms in the ITGB3 gene and asthma risk.

## Materials and Methods

### Ethical Statement

This study was approved by the Medical Ethics Committee of Shandong University, and written informed consent was obtained from the parents of every participant.

### Study Population

During the 6-year period from 2006 to 2012, 321 unrelated Chinese children with asthma were recruited as case subjects from the QiLu Children’s Hospital of Shandong University, with their parents’ consent; 315 unrelated healthy children were recruited randomly from Shandong province and the surrounding area as control subjects. The diagnosis of asthma in all subjects was performed by asthma specialists according to the modified criteria [5]: (1) recurrent wheezing, coughing, shortness of breath, and chest tightness, closely related to multiple factors, such as inhaled allergens, changes in weather, physical or chemical irritants, or respiratory tract infections, that frequently occur or worsen at night and/or in the early morning; (2) scattered lung wheezing sounds upon exhalation; (3) spontaneous remission of the above symptoms with anti-asthma treatment; and (4) the absence of other diseases that could cause wheezing, coughing, shortness of breath, and chest tightness.

### DNA Extraction

Each child contributed a 1 ml whole-blood sample to this study. Genomic DNA was extracted from the blood samples using a QIAamp DNA Mini Kit (QIAGEN). All DNA samples were quantified using a NANODROP 2000 spectrophotometer (Thermo) and stored at −20°C until use.

### SNP Selection and Primer Design

SNP genotype data for CHB were obtained from the HapMap database and analysed using Haploview software. SNPs were selected according to the following criteria: (1) r^2^ threshold of 0.8, as analysed by a pairwise tagging algorithm; (2) no C/G alleles; and (3) MAF>0.05, except for the functional SNP rs5918. Of the SNPs that met the above three criteria, a total of 6 SNPs were selected for genotyping: rs2015729 (Intron 2), rs2317676 (3′UTR), rs5918 (Exon 3), rs5919 (Exon 6), rs3809865 (3′UTR), and rs10514919 (Intron 1).

The primers used to amplify the selected SNPs were designed using Primer5 according to their flanking sequences, based on the following criteria: (1) melting temperature (Tm) between 60 and 65°C; (2) absence of dimerisation and mispriming capabilities; and (3) an amplicon size smaller than 100 bp, to ensure high sensibility [6].

Prior to HRM analysis, PCR conditions were confirmed to yield a single band to avoid interference from primer dimerisation and mispriming products.

### Genotyping

The PCR was performed in a final volume of 10 µL containing 25 ng DNA, 1.0 U Hot Start Taq-DNA polymerase (Takara), 1.25 µM of each primer, 0.8 µL dNTP MIX (Takara), 1.0 µL 10× PCR buffer (Takara) and 1.0 µL LC green. The amplification was carried out according to the following protocol: initial denaturation at 95°C for 5 min, followed by 30 cycles of 95°C for 30 s, annealing at 60°C for 30 s and elongation at 68°C for 30 s. All PCR amplifications were carried out in 96-well plates on a LightCycler 480 instrument (Roche) according to the manufacturer’s instructions. HRM curves were generated by monitoring the fluorescence of the sample during a temperature ramp from 65 to 95°C at 0.02°C/s. Normalised HRM curves were generated with the following normalisation regions: rs2015729, 82.22–83.33 and 89.85–90.74; rs2317676, 83.78–84.86 and 87.76–88.95; rs3809865, 82.01–83.57 and 87.6–88.99; rs5919, 81.6–83.2 and 86.42–88.47; rs5918, 82.15–84.22 and 89.79–91.44; and rs10514919, 83.04–84.48 and 88.41–90.23. HRM curves were classified into two or three distinct groups. Thirty random samples from each group were sequenced with an ABI 3100 Genetic Analyzer to confirm the accuracy of the genotyping. Samples with known genotypes were used as internal references to generate standard curves for the classification of the unknown samples.

### Luciferase Reporter Assay

A 227 bp region of ITGB3 3′UTR containing the putative recognition site for rs3809865 was amplified and ligated into pHSA-MIR-REPORT (Ambion). CRL1730 (human umbilical vein endothelial cells), 293T (human embryonic kidney cells), and A549 (human lung adenocarcinoma cells) were co-transfected with 400 ng of the 3′UTR-luciferase reporter vector and 20 nM mature hsa-mir-124 (final concentration) using Lipofectamine 2000 (Invitrogen). Control cells were transfected with 100 ng pRL-SV40 plasmid (Promega) for normalisation. After a 24 h incubation, luciferase and Renilla activities were measured using a Dual Luciferase Assay Kit (Promega) according to the manufacturer’s instructions.

### The Regulation of ITGB3 by hsa-mir-124

To further confirm the regulation of ITGB3 by hsa-mir-124, A549 was transfected with an hsa-mir-124 mimic (124-M, 5′UAAGGCACGCGGUGAAUGCCAAG3′) or a control miRNA (CON-M, 5′UAAccCACGCGGUGAAUGCCAAG3′). After a 40 h incubation, cells were collected, washed with ice-cold PBS and then lysed with RIPA lysis buffer (Cell Signaling Technology) in an ice bath for 5 min, followed by sonication for 8 s. The protein concentration of each sample was determined using a BCA protein assay kit (Pierce) according to the manufacturer’s protocol. Samples with equal amounts (35 µg) of protein were separated via 12% sodium dodecyl sulphate polyacrylamide gel electrophoresis (SDS-PAGE) and transferred to polyvinylidene fluoride membranes. The membranes were blocked for 1 h with 5% fat-free milk in Tris-buffered saline with 0.05% Tween-20 (TBST) and then incubated with the indicated primary antibodies overnight at 4°C. After washing three times with TBST, the membranes were incubated with horseradish peroxidase-conjugated secondary antibodies for 1 h at room temperature and washed three times with TBST. The labelled proteins were detected using the enhanced chemiluminescence method and quantified using Alpha Imager 2200.

Cells were fixed in 4% paraformaldehyde in PBS for 20 min at room temperature, rinsed in PBS and permeabilised with 0.5% Triton X-100 for 10 min. Subsequently, cells were blocked with 10% goat serum in PBST (PBS containing 0.1% Triton X-100). After three washes with PBST, the cells were incubated with rabbit polyclonal anti-ITGB3 primary antibody (1∶50, Abcam) overnight at 4°C, washed ten times with PBST and incubated with secondary antibody (1∶200, Anbo) for 1 h. The cells were then washed ten times with PBST and imaged under a fluorescence microscope (Leica).

### Statistical Analysis

Hardy-Weinberg equilibrium was evaluated using the Fisher’s exact test. The genotypic frequencies of all SNPs in cases and controls were compared by Chi-square (χ^2^) tests. The allele frequencies were compared by allelic associations test. Odds ratios with 95% confidence intervals (95% CI) and adjusted p values were calculated using allelic associations tests. The associations between genotypes and asthma were assessed using χ^2^ tests. Statistical analysis was performed in PLINK v1.07. P<0.05 was considered to be statistically significant.

## Results

In the present study, 321 paediatric asthma patients (female: 143, male: 178, median age: 6.6) and 315 controls (female: 131, male: 184, median age: 6.7) were recruited. Chi-square tests were used to assess whether the cases and controls were similar. As shown in Table 1, the case and control populations were well matched in terms of sex and age distributions.

**Table 1:** The basic characteristics of the cases and controls.

Variable		Case (N = 321)		Control (N = 315)		*p*-values from χ^2^
		No	%	No	%	
Sex	Female	143	44.55	131	41.59	0.75
	Male	178	55.45	184	58.41	
Age	2 to 4	35	10.9	30	9.52	0.84
	4 to 8	213	66.36	211	66.99	
	8 to 12	73	22.74	74	23.49	
	Median age	6.6		6.7		

### Detection of Amplicons

**Figure 1:**
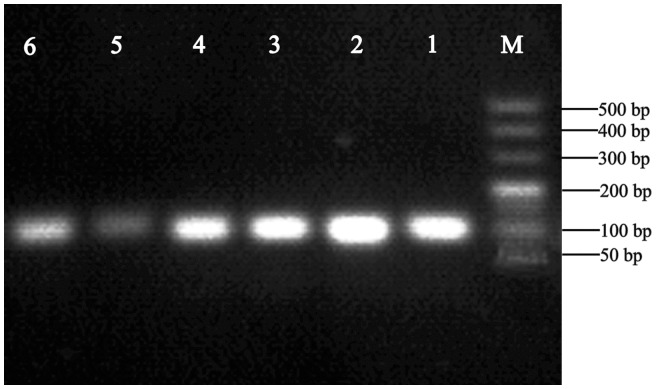
Analysis of amplicons by agarose electrophoresis. M, DNA maker DL500 (Takara); 1, rs10514919; 2, rs5919; 3, rs5918; 4, rs3859865; 5, rs2317676; 6, rs2015729.

The primers used to amplify the selected SNPs are listed in Table S1. PCR was performed under the aforementioned conditions, and the amplicons were analysed by 2% agarose electrophoresis. As shown in Fig. 1, each amplicon was unique and appeared at the expected size, indicating that the primers and PCR conditions were adequate for HRM analysis.

### Genotype Assignment

**Figure 2:**
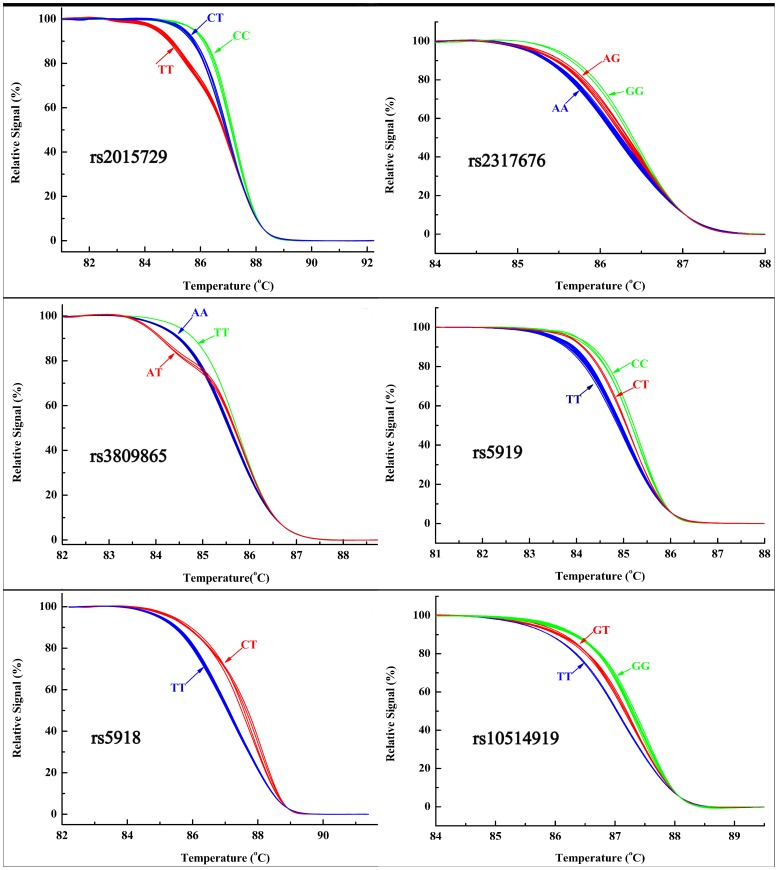
High resolution melting curves for different genotypes of six SNPs.

The genotype assignments of the selected SNPs were determined by HRM curves, using the sequenced samples as control genotypes. The studied SNPs were successfully genotyped by HRM analysis, as shown in Fig. 2. All of the tested SNPs were in Hardy-Weinberg equilibrium (HWE) in both the case and control groups (P>0.05). We first analysed the association between SNP genotypes and the risk of asthma. The frequency distributions of the genotypes are shown in Table 2. No differences in the genotype frequencies of the six SNPs were observed between the controls and cases (p>0.05 for all).

**Table 2:** Association between SNP genotypes and the risk of asthma.

SNP	Genotype	Frequency		Odds ratio(95% CI)	*p*-valuefrom χ^2^
		Case (%)	Control (%)		
rs2015729	CC	43 (13.69)	51(16.56)	1 ref	0.55
	CT	160 (50.96)	165(53.57)	1.15 (0.73–1.83)	
	TT	111 (35.35)	92 (29.87)	1.43 (0.88–2.34)	0.15
rs2317676	AA	197 (61.75)	198 (64.5)	1.266(0.56–2.86)	0.57
	GA	111 (34.8)	95 (30.94)	1.487(0.64–3.43)	0.35
	GG	11 (3.45)	14 (4.56)	1 ref	
rs3809865	AA	242 (77.99)	201(65.47)	2.11(0.87–5.12)	0.09
	AT	62 (19.5)	92(29.97)	1.18(0.47–2.98)	0.73
	TT	8 (2.52)	14 (4.56)	1 ref	
rs5918	TT	301 (95.25)	296(96.73)	1 ref	
	CT	15 (4.75)	10 (3.27)	2.63 (1.24–5.54)	0.348
	CC	0 (0)	0 (0)	NA	
rs5919	TT	183 (57.01)	180 (57.32)	1 ref	
	CT	114 (35.51)	121 (38.54)	0.93 (0.67–1.29)	0.65
	CC	24 (7.48)	13 (4.14)	1.82 (0.9–3.68)	0.09
rs10514919	GG	198 (62.26)	193 (61.86)	1 ref	
	GT	99 (31.13)	108 (34.61)	0.89 (0.64–1.25)	0.51
	TT	21 (6.61)	11 (3.53)	1.86 (0.87–3.96)	0.1

CI, confidence interval.

We further compared the frequency distributions of the minor alleles between the controls and cases using χ^2^ tests (Table 3). The minor allele (C) of rs5918 was present at a slightly higher frequency in the case group (MAF = 0.024) than in the control group (MAF = 0.016), with an odds ratio of 1.46 (0.65–3.28), but it showed no association with asthma (p>0.05). The other four SNPs, including rs2015279, rs2317676, rs5919, and rs10514919, were also well genotyped but showed no significant difference between controls and cases (p>0.05 for each).

**Table 3:** Minor allele frequencies of six selected SNPs in cases and controls and association with asthma.

SNP	Location ingene	Position	Minorallele	MAFCase	MAFControl	*p*-valuesfrom χ^2^	adjusted*p*-values	Odds ratio(95% CI)
rs2015729	Intron 2	42709492	C	0.39	0.43	0.14	0.81	0.84 (0.67–1.06)
rs2317676rs3809865	3′UTR3′UTR	4274328242743585	GT	0.210.13	0.20.2	0.720.0007	10.004	1.05 (0.8–1.38)0.59 (0.43–0.8)
rs5918rs5919rs10514919	Exon 3Exon 6Intron 1	427157294271953942697128	CCT	0.0240.250.22	0.0160.230.21	0.3530.450.56	111	1.46 (0.65–3.28)1.1 (0.85–1.43)1.08 (0.83–1.42)

MAF, minor allele frequency; CI, confidence interval. Adjusted *p*-values were obtained by Bonferroni correction.

The SNP within the 3′UTR of ITGB3, rs3809865, showed a significantly different distribution between cases and controls (p = 0.0007). The frequency of the minor allele (T) was obviously higher in the control group than in the case group (MAF = 0.2 and 0.13, respectively). The odds ratio (95% CI) was 0.59 (0.43–0.8) using the major allele (A) as a reference, which suggested that this allele provided some protection again asthma. After Bonferroni correction, rs3809865 still showed a significant association with asthma (p = 0.004).

### Targeting of ITGB3 by hsa-mir-124

**Figure 3:**
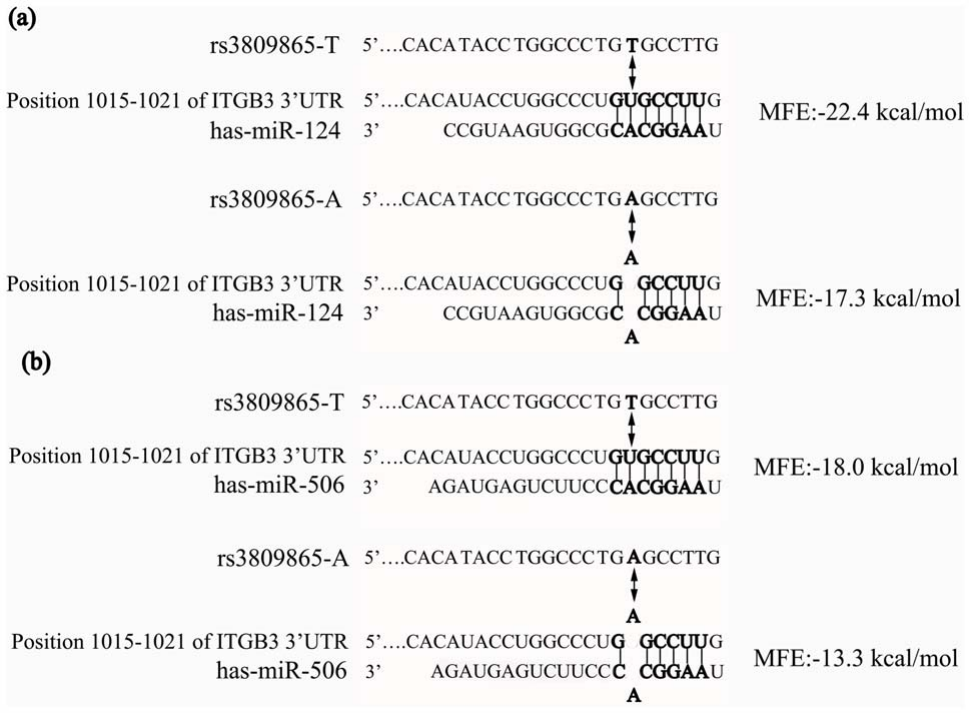
Predicted binding of hsa-mir-124 and hsa-mir-506 to 3′UTR of ITGB3. The bold letters represented the predicted consequential pairing of target region and miRNA. Rs3809865-T represented T allele of this SNP and rs3809865-A represented A allele. The two-way arrow indicated the location of rs3809865 in ITGB3. The minimum free energy (MFE) were calculated by RNAhybrid (http://bibiserv.techfak.uni-bielefeld.de/rnahybrid/).

We used TargetScan (http://www.targetscan.org/) and PITA (http://genie.weizmann.ac.il/pubs/mir07/mir07_data.html) to identify miRNAs that were predicted to bind to the 3′UTR of ITGB3. Two miRNAs (hsa-mir-124 and hsa-mir-506) were found to have seed regions that bound to the sequence containing rs3809865 (Fig. 3). These results suggested that these two miRNAs should bind more stably to the T allele of rs3809865 than to the A allele. Thus, it was deduced that the allele of rs3809865 might affect the miRNA binding to ITGB3 and thus regulate the expression of ITGB3.

**Figure 4:**
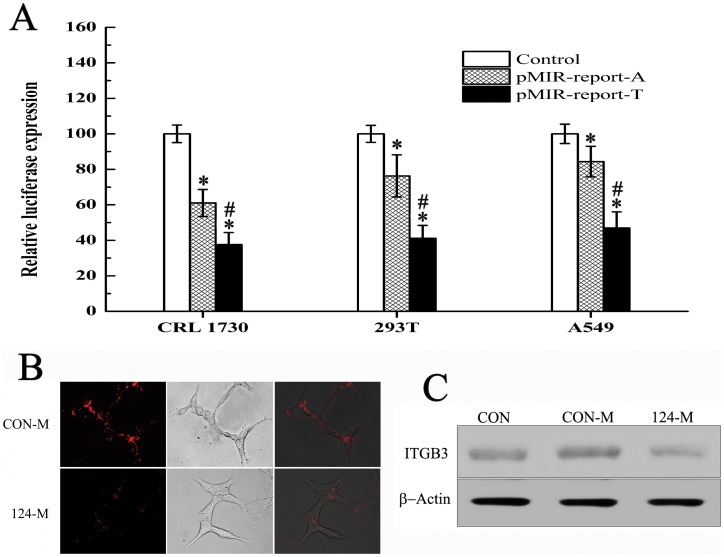
The effect of rs3809865 on the targeting of hsa-mir-124 and the expression of ITGB3. (A) Luciferase reporter assays suggesting the allele-specific targeting of hsa-mir-124 to rs3809865 in CRL1730, 293T and A549 cells. *, p<0.05 vs.control group, ^#^, p<0.05 vs. pHSA-MIR-report-A group. Each transfection was performed at least five times. (B) and (C) The regulation of ITGB3 expression by transfecting hsa-mir-124 in A549 cells analyzed by immunocytochemistry staining and Western blot. CON-M represented control miRNA and 124-M represented hsa-mir-124 mimic.

Luciferase assays were performed to validate this prediction. As shown in Fig. 4A, hsa-mir-124 significantly inhibited the expression of the luciferase gene from the vector carrying the rs3809865 T allele compared with the vector carrying the rs3809865 A allele and the control vector in all cell lines tested (CRL1730∶37.6% expression for the T allele versus 61% for the A allele; 293T: 41.1% expression for the T allele versus 76.3% for the A allele; A549∶46.9% expression for the T allele versus 84.4% for the A allele). Having established that hsa-mir-124 could interact with this putative binding site, we further investigated the regulation of ITGB3 by hsa-mir-124. The results showed that the transfection of A549 with hsa-mir-124 resulted in an obvious decrease in expression of ITGB3, as analysed by immunofluorescence and western blotting.

## Discussion

In the present study, HRM technology was applied to genotype the selected SNPs in the ITGB3 gene. Six SNPs were accurately genotyped using HRM. The genotyping was especially successful for rs3809865 with the similar GC content, which suggested that small amplicons were good substrates for SNP genotyping by the HRM method.

Based on the HRM genotyping results, the associations between the six SNPs in ITGB3 gene and asthma were investigated in Chinese Han children. Previous studies have demonstrated that the ITGB3 gene plays an important role in asthma pathogenesis. Among the tested SNPs in ITGB3, the minor allele of rs2015729 exhibited a strong association with asthma in a Hutterite population (0.001<p<0.01), and the major allele showed modest association in a population from Madison (0.01<P<0.05), but this association was not replicated in populations from Chicago [2]. Our results suggested that this SNP was not associated with asthma in Chinese Han children. A modest association between rs2317676 and asthma has been identified in a Caucasian population from Chicago (0.01<P<0.05) [2]. Furthermore, rs2317676 was correlated with IgE level in a group of children from Madison and was also associated with asthma in their parents [1]. In the present investigation, however, rs2317676 showed no association with asthma. The coding variant rs5918 has shown a weak association with wheezing and asthma in the Madison population [1], but no relationship was observed in this investigation.

The rs3809865 allele was strongly associated with asthma in Chinese Han children. This SNP tags an asthma-associated SNP (rs3760372, 0.001<P<0.01) with an r^2^ value of 1.0 [2], which seems likely to account for this result. The SNPs located in the 3′UTRs of genes have attracted attention due to their roles in regulating gene expression. Our TargetScan and PITA analyses indicated that rs3809865 might influence miRNA binding to ITGB3. The sequence containing the T allele of rs3809865 bound more stably to hsa-mir-124 and hsa-mir-506 than the sequence containing the A allele, which would likely result in the down-regulation of integrin β3. It has been reported that decreases in integrin β1 and β4 levels could prevent the development of asthma [6], [7], but it is unclear whether the down-regulation of integrin β3 could have a similar effect. Therefore, we further investigated the regulation of integrin β3 by hsa-mir-124. Luciferase reporter assays suggested that the allele of rs3809865 could affect the targeting of hsa-mir-124. Subsequently, it was confirmed that the expression of integrin β3 in A549 cells was suppressed by transfection with hsa-mir-124, which indicated that hsa-mir-124 could regulate the expression of integrin β3. These results clarify the mechanism underlying the association between rs3809865 and the decreased risk of asthma.

In this study, we evaluated three SNPs that have been associated with asthma in Western populations but failed to replicate these associations in Chinese Han children. The different findings in distinct populations might be due to differences in environmental conditions. Environmental exposures have been shown to have important roles in triggering asthma [8]. Various environmental factors, such as air pollution, microbial exposure, diet and pet ownership, could affect asthma development [9]–[12]. Our subjects lived mainly in Jinan city and the surrounding area and thus were subjected to different environmental risk factors than their Western counterparts, likely explaining the difference in results. Other differences between studies, such as the criteria used to define asthma, the age and race of the subjects and differences in the patterns of linkage disequilibrium are also factors that might explain non-replication in genetic association studies [3].

Overall, the findings presented in this study provide new evidence for the association between SNPs in ITGB3 and asthma. Our results, combined with those of previous reports, suggested that ITGB3 should be considered a true asthma-related gene.

## Supporting Information

Table S1 Sequences and T_m_ values of Primers and product sizes
(DOCX)
