# Supplementary material for: Genetic Variation of ITGB3 Is Associated with Asthma in Chinese Han Children
Source: PLoS One. 2013 Feb 22;8(2):e56914. doi: 10.1371/journal.pone.0056914 (PMC3579922; doi:10.1371/journal.pone.0056914)
Supplement: Table S1 — Sequences and Tm values of Primers and product sizes (DOCX) [file pone.0056914.s001.docx]

**Luciferase reporter assay**

A 227 bp region of ITGB3 3’UTR containing the putative recognition site for rs3809865 was amplified from DNA samples with the A and T alleles, respectively, using forward (5’ TCTCGAGCTCTCAAAGGGAGAGAGTGCTATT3’) and reverse (5’ TCTCAAGCTTTACAACTGACCCATCATTCCA3’) primers containing *Sac*I and *Hind*III restriction sites. The PCR products were digested and ligated into pHSA-MIR-REPORT (Ambion). CRL1730 (human umbilical vein endothelial cells), 293T (human embryonic kidney cells), and A549 (human lung adenocarcinoma cells) were co-transfected with 400 ng of the 3’UTR-luciferase reporter vector and 20 nM mature hsa-mir-124 (final concentration) using Lipofectamine 2000 (Invitrogen). Control cells were transfected with 100 ng pRL-SV40 plasmid (Promega) for normalisation. After a 24 h incubation, luciferase and Renilla activities were measured using a Dual Luciferase Assay Kit (Promega) according to the manufacturer’s instructions.

**Table S1 Sequences and T_m_ values of Primers and product sizes**

| SNP | Primer sequence (5’- 3’) | T_m_ | PCR product size (bp) |
| --- | --- | --- | --- |
| rs2015729 | F : CAGAGAGAGCACAGCAACCCA  R: GGATGAGCACGGAGTTTTGG | 61.0  60.2 | 90 |
| rs5918 | F:CTT CTCTTTGGGCTCCTGTCTTAC  R: CATTCTGGGGCACAGTTATCCTT | 60.9  62.3 | 97 |
| rs3809865 | F: CTCCTGTCCCTCATCCATAGCAC  R: TAGAAGGTAAATACAATCAGCCCCA | 62.5  61.7 | 92 |
| rs2317676 | F: CTAATTCTTTGACCTGTTGGGAGTG  R: GTGATTATGAACTGGGAGATGCTG | 61.5  60.6 | 96 |
| rs5919 | F: GGTGTTTACCACTGATGCCAAGACT  R: ACTACCAACATGACACTGCCCG | 63.6  62.2 | 94 |
| rs10514919 | F : TTGGGTTGTTTCTGGAATGTCTG  R: GAAAAGGTCTCTCTGTGGAGCTGA | 60.9  61.9 | 94 |

T_m_, melting temperature.
